# Ginsenoside Re Promotes Osteoblast Differentiation in Mouse Osteoblast Precursor MC3T3-E1 Cells and a Zebrafish Model

**DOI:** 10.3390/molecules22010042

**Published:** 2016-12-29

**Authors:** Hye-Min Kim, Dong Hyun Kim, Ho-Jin Han, Chan-Mi Park, Srinivas Rao Ganipisetti, Mariadhas Valan Arasu, Young Ock Kim, Chun Geun Park, Bo-Yeon Kim, Nak-Kyun Soung

**Affiliations:** 1 Anticancer Agent Research Center, Korea Research Institute of Bioscience and Biotechnology (KRIBB), Cheongju 28116, Korea; hyemin88@kribb.re.kr (H.-M.K.); waikiki@kribb.re.kr (D.H.K.); hjhan@kribb.re.kr (H.-J.H.); raogs@kribb.re.kr (S.R.G.); 2 World Class Institute, Korea Research Institute of Bioscience and Biotechnology (KRIBB), Cheongju 28116, Korea; springcm@kribb.re.kr; 3 Department of Botany and Microbiology, Addiriyah Chair for Environmental Studies, College of Science, King Saud University, P.O. Box 2455, Riyadh 11451, Saudi Arabia; mvalanarasu@gmail.com; 4 Department of Medicinal Crop Research Institute, National Institute of Horticultural & Herbal Science, Rural Development Administration, Eumseong 27709, Korea; kyo9128@korea.kr (Y.O.K.); pcg@korea.kr (C.G.P.); 5 Department of Biomolecular Science, University of Science and Technology, Daejeon 34113, Korea

**Keywords:** bone, osteoblast differentiation, Ginsenoside Re

## Abstract

Bone homeostasis is tightly regulated to balance bone formation and bone resorption. Many anabolic drugs are used as bone-targeted therapeutic agents for the promotion of osteoblast-mediated bone formation or inhibition of osteoclast-mediated bone resorption. Previous studies showed that ginsenoside Re has the effect of the suppression of osteoclast differentiation in mouse bone-marrow derived macrophages and zebrafish. Herein, we investigated whether ginsenoside Re affects osteoblast differentiation and mineralization in in vitro and in vivo models. Mouse osteoblast precursor MC3T3-E1 cells were used to investigate cell viability, alkaline phosphatase (ALP) activity, and mineralization. In addition, we examined osteoblastic signaling pathways. Ginsenoside Re affected ALP activity without cytotoxicity, and we also observed the stimulation of osteoblast differentiation through the activation of osteoblast markers including runt-related transcription factor 2, type 1 collagen, ALP, and osteocalcin in MC3T3-E1 cells. Moreover, Alizarin red S staining indicated that ginsenoside Re increased osteoblast mineralization in MC3T3-E1 cells and zebrafish scales compared to controls. These results suggest that ginsenoside Re promotes osteoblast differentiation as well as inhibits osteoclast differentiation, and it could be a potential therapeutic agent for bone diseases.

## 1. Introduction

Bone is the most important tissue in the body skeleton. Bones preserve the various organs, regulate blood calcium, produce hematopoietic cells, and support the body. The bone remodeling refers to the repeated processes of bone formation and bone resorption, the former using osteoblasts and the letter osteoclast, in order to maintain a healthy skeleton [1,2]. An imbalance in bone homeostasis leads to metabolic diseases such as osteoporosis, osteopetrosis, and Paget’s diseases. Osteoporosis is a common metabolic disorder related to bone remodeling, which causes the collapse of bone homeostasis, leading to a loss of bone mass and a consequent bone fragility. For the treatment of osteoporosis, estrogen, bisphosphonates, calcitonin, and selective estrogen receptor modulators are now on the market [3,4]. However, these drugs have many side effects such as osteonecrosis of the jaw (ONJ), atrial fibrillation, and esophageal cancer [5,6,7,8].

Osteoblasts are derived from mesenchymal stem cells (MSCs), which specialize in bone formation. The cells express osteoblastogenic factors, leading to the formation of bone matrix proteins, and bone mineralization [1,9]. Osteoblast differentiation is an important step in bone formation and is regulated by runt-related transcription factor 2 (Runx2) and osterix (Osx), which are key transcription factors in osteoblast differentiation [10]. Moreover, Runx2 regulates several osteoblastic marker genes such as type 1 collagen (Col 1), alkaline phosphatase activity (ALP), and osteocalcin (Ocn) [11].

**Figure 1:**
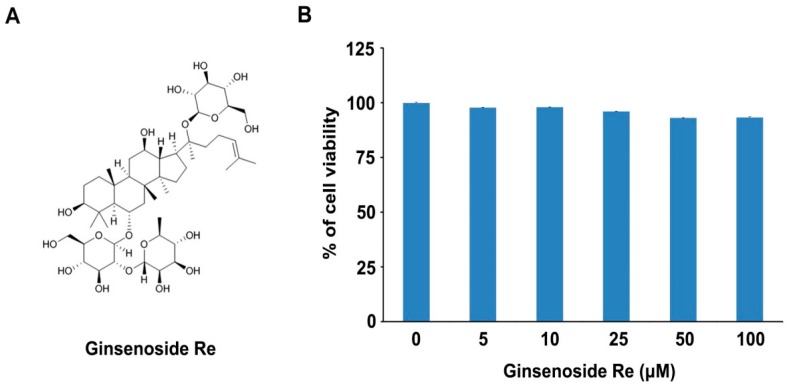
Chemical structure of (**A**) ginsenoside Re and (**B**) the effect of ginsenoside Re on the cell viability in MC3T3-E1 cells. The cells were treated and incubated with ginsenoside Re (5, 10, 25, 50, and 100 μM concentrations) for 24 h and cell viability was measured via MTT assay.

*Panax gineseng* (Korean ginseng) is commonly used for medical purposes as an anti-inflammation, cancer prevention, and erectile dysfunction [12,13,14]. Ginsengs contain various active components including ginsenosides, polysaccharides, peptides, polyacetylenic alcohols, and fatty acids. Among them, ginsenosides are the major components in an aspect of pharmacological effects of ginseng [15]. To date, more than 150 naturally-occurring ginsenosides have been identified. Among the ginsenosides, ginsenoside Re is a major ginsenoside and is soluble in water (Figure 1A). Ginsenoside Re is extracted from ginseng root, and it is more in wild ginseng roots than in cultivated ones. Ginsenoside Re exhibits a multiple of pharmacological activities including anti-diabetic and anti-oxidative effects, and cardiovascular system support [16,17,18,19]. We recently have reported that ginsenoside Re influences bone remodeling through the inhibition of osteoclast differentiation [20].

In this study, we investigated the effects of ginsenoside Re on osteoblast differentiation and mineralization. We observed that ginsenoside Re increased alkaline phosphatase (ALP) activity in MC3T3-E1 cells in vitro and promoted mineralization in MC3T3-E1 cells and zebrafish scales in vivo.

## 2. Results and Discussion

### 2.1. Effects of Ginsenoside Re on Cell Viability in Mouse Osteoblast Precursor MC3T3-E1 Cells

An appropriate balance of osteoblasts and osteoclasts is important in bone remodeling; impaired bone homeostasis can cause bone diseases such as bone fracture and osteoporosis [3,4]. Ginseng has been used for the treatment of bone diseases. The effects of Korean red ginseng were reported about radiation-induced bone loss, glucocorticoid-induced osteoporosis, receptor activator of nuclear factor-κB ligand (RANKL) induced osteoclast differentiation, and estrogen deficiency-induced osteoporosis [21,22,23]. Among the ginseng components, ginsenosides, also known as ginseng saponins, have been identified to be therapeutic agents via the inhibition of osteoclasts or the promotion of osteoblasts. Ginsenoside Rb1, Rg1, and Rg3 inhibit osteoclast differentiation [24,25,26]. Moreover, ginsenoside Rg5, Rk1, Rh1, and Rd promote osteoblast differentiation [27,28,29,30]. Moreover, ginsenoside Rh2 has dual functions: the inhibition of osteoclast differentiation and the promotion of osteoblast differentiation [31,32,33].

Our previous study demonstrated that ginsenoside Re inhibits osteoclast differentiation in mouse bone marrow-derived macrophages (BMMs) and zebrafish [20]. In order to test whether ginsenoside Re has the osteoblast promoting function, we investigated cell viability in MC3T3-E1 cells, which have been used to test osteoblast differentiation. MC3T3-E1 cells were cultured in the presence of ginsenoside Re (5, 10, 25, 50, and 100 μM concentrations). In 36 h, cytotoxicity was measured using a MTT assay. We observed that the proliferation of MC3T3-E1 cells was not diminished even with 100 μM ginsenoside Re (Figure 1B). Therefore, we concluded that ginsenoside Re has no cytotoxicity in MC3T3-E1 cells.

### 2.2. Effects of Ginsenoside Re on ALP Activity in Mouse MC3T3-E1 Cells

**Figure 2:**
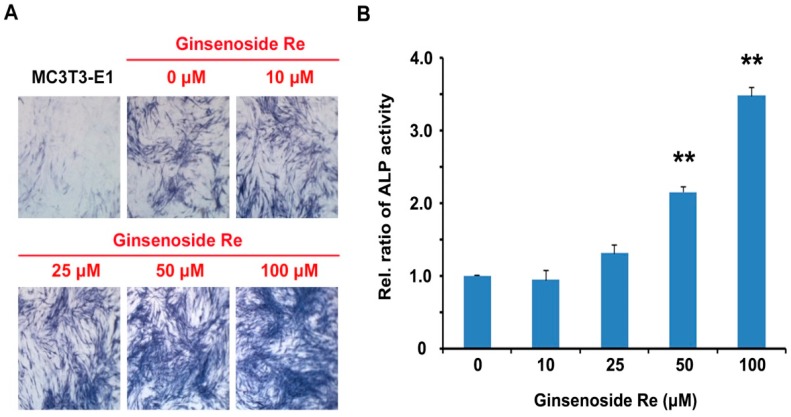
The effect of ginsenoside Re on alkaline phosphatase (ALP) (**A**) staining and (**B**) activity in MC3T3-E1 cells. The cells were cultured in osteogenic medium for 14 days; staining and activity of ALP was performed using BCIP/NBT and *p*NPP as a substrate. All experiments were performed in triplicate. ** *P* < 0.01 vs. the control.

Osteoblasts are bone-forming cells that express diverse osteoblastic markers including ALP activity, collagens, non-collagenous matrix proteins (NCPs), and osteocalcin [34,35]. ALP is considered the most relevant biochemical marker in osteoblast differentiation and maturation. Activity and localization of ALP are necessary for bone development and differentiation [36,37]. Therefore, we evaluated the effects of ginsenoside Re on ALP activity in MC3T3-E1 cells. The cells were cultured in an osteogenic medium with and without ginsenoside Re for 14 days, respectively. ALP staining and activity were measured by its enzymatic activity with *p*NPP as a substrate. The cells treated with ginsenoside Re exhibited dose-dependent promotion of ALP-positive staining in comparison to those without ginsenoside Re (Figure 2A). ALP activity was also stimulated ginsenoside Re with concentrations between 50 and 100 μM (Figure 2B). Although the concentration of ginsenoside Re in promoting osteoblast differentiation was 10–20 times higher than that of osteoclast differentiation inhibition. These results were similar to other ginsenosides such as ginsenoside Rd (~40 μM) and ginsenoside Rh1 (10~100 μM) [28,29]. These findings suggest that ginsenoside Re promotes osteoblast differentiation.

### 2.3. Effects of Ginsenoside Re on mRNA Expression of Osteoblast Differentiation Markers in MC 3T3-E1 Cells

**Figure 3:**
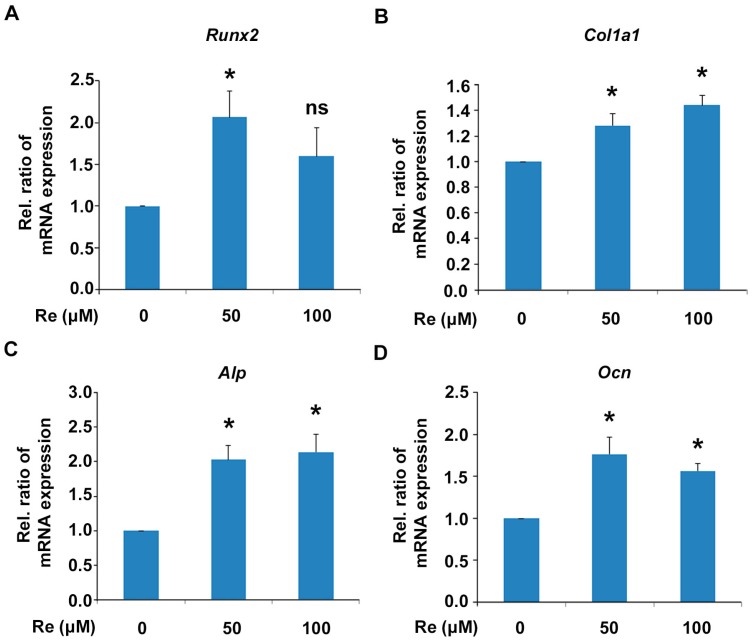
Effects of ginsenoside Re on mRNA expression level of osteoblast marker genes in MC3T3-E1 cells. The cells were cultured for 7 days in the presence or absence of ginsenoside Re. Then, quantitative RT-PCR analysis of (**A**) *Runx2*; (**B**) *Col1a1* (type 1 collagen); (**C**) *Alp*; and (**D**) *Ocn* (Osteocalcin) mRNA expression was performed in MC3T3-E1 cells. The results were normalized by the mRNA level of GAPDH. All experiments were performed in triplicate. * *P* < 0.05 vs. the control.

We further investigated the effects of ginsenoside Re on mRNA expression of osteoblast differentiation marker genes using quantitative reverse transcription-polymerase chain reaction PCR (qRT-PCR). MC3T3-E1 cells were incubated in osteogenic medium in the presence of ginsenoside Re for 7 days, and the total RNA was extracted for qRT-PCR. The mRNA level of *Runx2*, a key transcription factor in osteoblast differentiation, was increased at 50 μM ginsenoside Re (Figure 3A). Ginsenoside Re treatment also increased the mRNA levels of osteoblast markers *Col1a1*, *Alp*, and *Ocn* in MC3T3-E1 cells (Figure 3B–D). These results suggest that ginsenoside Re stimulates osteoblast differentiation through Runx2 and the stimulation of downstream osteoblast marker genes.

### 2.4. Effects of Ginsenoside Re on Mineralization in MC3T3-E1 Cells and Zebrafish Scales

**Figure 4:**
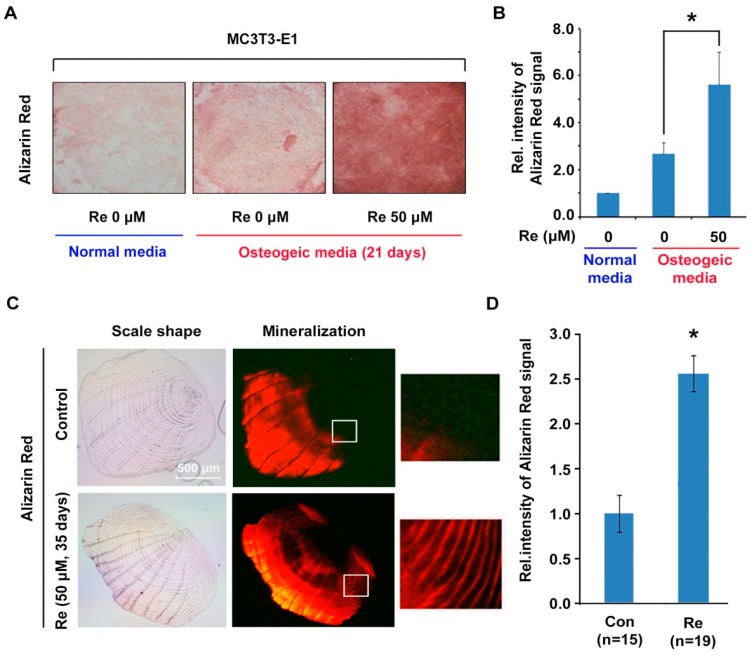
Effects of ginsenoside Re on mineralization of MC3T3-E1 cells and zebrafish scales. (**A**) MC3T3-E1 cells were treated with ginsenoside Re for 21 days and stained with Alizarin red S to visualize calcium deposition; (**B**) The measurement of intensity of Figure 4A, normalized by normal media treated sample intensity. All experiments were performed in triplicate, and the intensity was measured by ImageJ tools. The sample in normal media was used as control; (**C**) The zebrafish were treated with ginsenoside Re for 35 days and their scales were stained with Alizarin red S to visualize calcium deposition; (**D**) The measurement of intensity of Figure 4C, normalized by control sample intensity. The intensity was measured by ImageJ tools. * *p* < 0.05 vs. the control.

The bone is the most relevant mineralized tissue in the body. Bone mineralization is frequently used as a marker to characterize osteoblast differentiation [11]. We examined the effects of ginsenoside Re on mineralization in MC3T3-E1 cells. The cells were incubated with ginsenoside Re in osteogenic medium for 21 days, and mineralization assay was carried out using Alizarin red S staining. We observed that calcium deposits were increased in MC3T3-E1 cells in ginsenoside Re at a 50 μM concentration (Figure 4A,B).

To determine the in vivo effects of ginsenoside Re on osteoblast mineralization, a zebrafish model was used. The zebrafish model is a valuable animal model to study bone development because the bone architecture and genetics of zebrafish are similar to those of humans. The zebrafish scale structure is very similar to human woven bone, and scleroblast as a scale-forming cell has a functional similarity to human osteoblast [38,39,40]. Recently, the fish scale has been applied to measurements of scleroblasts and osteoclasts activity [41,42]. Zebrafish were raised in water containing 50 μM ginsenoside Re for 35 days, and their scales were stained with Alizarin red S solution. Zebrafish scales treated with ginsenoside Re showed increased calcium concentrations through the relative intensity of Alizarin red S. This signal intensity was 2.4-fold higher than that of control scales (Figure 4C,D). Taken together, we suggest that ginsenoside Re promotes osteoblast mineralization both in vitro and in vivo.

## 3. Materials and Methods

### 3.1. Chemicals

Ginsenoside Re as the analytical standard was obtained from Sigma Aldrich (St. Louis, MO, USA). l-ascorbic acid and β-glycerophosphate as osteoblastic factors were also purchased from Sigma Aldrich.

### 3.2. Cell Culture of MC3T3-E1 Cells

Mouse osteoblast precursor cell line MC3T3-E1 was maintained in an alpha modification of Eagle’s minimum essential medium (α-MEM) (Gibco, Grand Island, NY, USA) supplemented with 10% fetal bovine serum (FBS) (Hyclone, CA, USA) and 1% penicillin-streptomycin. To induce osteoblast differentiation, MC3T3-E1 cells (5 × 10^3^ cells/well) were seeded onto a 96-well plate and cultured with osteogenic medium containing 100 μg/mL of L-ascorbic acid and 10 mM of β-glycerophosphate as osteogenic factors.

### 3.3. MTT Assay

Cell viability assay was measured using a 3-(4,5-dimethylthiazol-2-yl)-2,5-diphenyltetrazolium bromide (MTT) assay (DoGenBio, Seoul, Korea). MC3T3-E1 cells were seeded at 5 × 10^3^ cells/well on a 96-well plate and incubated for 24 h. The cells were treated with various concentrations of ginsenoside Re (5, 10, 25, 50, and 100 μM) for 36 h. After incubation, the MTT solution was added for 2 h. Finally, cell viability was monitored at 450 nm using a plate reader.

### 3.4. Alkaline Phosphatase (ALP) Enzyme Staining and Activity

Staining of ALP activity was performed with BCIP/NBT substrate solution (Sigma Aldrich), according to the manufacturer’s instructions. MC3T3-E1 cells were incubated with osteogenic medium for 14 days in the presence of ginsenoside Re. The cells were fixed with 10% formalin and stained with BCIP/NBT substrate solution in the dark for 10 min. ALP activity was also measured using the diethanolamine detection kit (Sigma Aldrich). The cells were washed with PBS and then lysed with 0.1% Triton-X100 in PBS. The cell lysates were centrifuged at 15,000 rpm for 10 min at 4 °C. The supernatant was incubated with *p*-nitrophenyl phosphate (*p*NPP) substrate solution for 1 h at 37 °C. The reaction was measured at 405 nm using a spectrophotometer.

### 3.5. Mineralization Analysis

MC3T3-E1 cells were seeded at 5 × 10^3^ cells/well in 96-well plate and incubated for 21 days in the presence or in absence of ginsenoside Re; the media was changed every 3 days. Cells were washed with PBS and fixed with 10% formalin for 30 min, and the cells were then stained with 40 mM Alizarin red S solution for 1 h. Zebrafish scales, which were separated from the body, were stained with Alizarin red S, as described elsewhere [38]. The scales were fixed with formaldehyde and were stained with Alizarin red (20 μg/mL, pH 4.1) dissolved in a 1% KOH solution.

### 3.6. Quantitative Real-Time RT-PCR

Total RNA was isolated from MC3T3-E1 cells with or without ginsenoside Re using an RNeasy mini kit (Qiagen, Valencia, CA, USA), and cDNA was synthesized using M-MLV reverse transcriptase (Promega, Fitchburg, WI, USA) according to the manufacturer’s instructions. Specific primers including *Col1a1*, *Alp*, *Ocn*, and *Runx2* were used for osteoblast marker genes as described previously [43]. The expression level of the genes was normalized to *GAPDH* transcript levels.

### 3.7. Zebrafish Housing 

All experimental procedures followed our previous study [20]. In brief, wild-type adult zebrafish were raised at 27.0 ± 1.0 °C under a 14:10 h light/dark cycle. Ten zebrafish were separated as a group into standard tanks and fed with live artemia stored in water. The water was replaced with 50% fresh water with or without ginsenoside Re (50 μM) every day. In 35 days, the fish were anesthetized with 0.01% tricaine methanesulfonate (Sigma Aldrich). The scales were then carefully removed from either side of the body using forceps.

### 3.8. Statistical Analysis

All data are presented as the mean ± SD of the three experiments. Data were analyzed via one-way analysis of variance (ANOVA) followed by Dunnett's multiple comparisons test. A value of *p* less than 0.05 was considered statistically significant.

## 4. Conclusions

This is the first study investigating the stimulation of osteoblast differentiation and mineralization by ginsenoside Re. Ginsenoside Re not only increased ALP activity, but also stimulated mRNA expression of osteoblastic markers *Runx2*, *Col1a1*, *Alp,* and *Ocn* without cytotoxicity. Moreover, ginsenoside Re increased osteoblast mineralization in mouse osteoblast precursor MC3T3-E1 cells and zebrafish scales. Therefore, our findings suggest that ginsenoside Re promotes osteoblast differentiation to improve bone health. Furthermore, ginsenoside Re have dual effects through the inhibition of osteoclast differentiation and the promotion of osteoblast differentiation. Our results indicate that ginsenoside Re was efficient for the prevention of bone weakness because of its dual functions. We propose that ginsenoside Re could be an important bone health supplement.
